# A Comparative Antibody Analysis of Pannexin1 Expression in Four Rat Brain Regions Reveals Varying Subcellular Localizations

**DOI:** 10.3389/fphar.2013.00006

**Published:** 2013-02-06

**Authors:** Angela C. Cone, Cinzia Ambrosi, Eliana Scemes, Maryann E. Martone, Gina E. Sosinsky

**Affiliations:** ^1^National Center for Microscopy and Imaging Research, Center for Research in Biological Systems, University of CaliforniaSan Diego, La Jolla, CA, USA; ^2^Dominick P. Purpura Department of Neuroscience, Albert Einstein College of Medicine, Pelham Parkway Albert Einstein College of MedicineBronx, NY, USA; ^3^Department of Neurosciences, University of CaliforniaSan Diego, La Jolla, CA, USA

**Keywords:** purinergic receptors, pannexin channels, ATP signaling, large field mosaic fluorescent imaging, paracrine signaling, connexin, knockout mouse

## Abstract

Pannexin1 (Panx1) channels release cytosolic ATP in response to signaling pathways. Panx1 is highly expressed in the central nervous system. We used four antibodies with different Panx1 anti-peptide epitopes to analyze four regions of rat brain. These antibodies labeled the same bands in Western blots and had highly similar patterns of immunofluorescence in tissue culture cells expressing Panx1, but Western blots of brain lysates from Panx1 knockout and control mice showed different banding patterns. Localizations of Panx1 in brain slices were generated using automated wide field mosaic confocal microscopy for imaging large regions of interest while retaining maximum resolution for examining cell populations and compartments. We compared Panx1 expression over the cerebellum, hippocampus with adjacent cortex, thalamus, and olfactory bulb. While Panx1 localizes to the same neuronal cell types, subcellular localizations differ. Two antibodies with epitopes against the intracellular loop and one against the carboxy terminus preferentially labeled cell bodies, while an antibody raised against an N-terminal peptide highlighted neuronal processes more than cell bodies. These labeling patterns may be a reflection of different cellular and subcellular localizations of full-length and/or modified Panx1 channels where each antibody is highlighting unique or differentially accessible Panx1 populations. However, we cannot rule out that one or more of these antibodies have specificity issues. All data associated with experiments from these four antibodies are presented in a manner that allows them to be compared and our claims thoroughly evaluated, rather than eliminating results that were questionable. Each antibody is given a unique identifier through the NIF Antibody Registry that can be used to track usage of individual antibodies across papers and all image and metadata are made available in the public repository, the Cell Centered Database, for on-line viewing, and download.

## Introduction

One mechanism of paracrine cell–cell communication occurs by ATP release and signal transduction through purinergic receptors (Burnstock, 2011). In the nervous system, ATP signaling stimulates neurotransmission, neuromodulation, and secretion as well as playing a role in cell proliferation, differentiation, and inflammation (Fields and Stevens, 2000). Membrane bound purinergic receptors are found on the plasma membrane of neurons and non-neuronal cells such as astrocytes and microglia (Fields and Stevens, 2000). Several studies have shown an association of the purinergic receptors with pannexin1 (Panx1), a connexin-like protein, which when stimulated acts as an ATP release channel (Locovei et al., 2006b; Nishida et al., 2008; Silverman et al., 2009; Kim and Kang, 2011; Poornima et al., 2011; Vessey et al., 2011). *In situ* hybridization imaging demonstrated high expression levels of Panx1 mRNA in the central nervous system (Ray et al., 2005; Vogt et al., 2005).

Panx1 has been proposed to fulfill a function in adaptive/inflammation responses following specific stimuli (Sosinsky et al., 2011). Panx1 channels have been shown to release ATP during gustatory channel response in taste bud cells (Romanov et al., 2007), the activation of the immune response in macrophages (Pelegrin and Surprenant, 2006), T lymphocytes (Schenk et al., 2008), and neurons (Silverman et al., 2009), pressure overload-induced fibrosis in the heart (Nishida et al., 2008) and NMDA receptor epileptiform electrical activity in the hippocampus (Thompson et al., 2008). This signaling pathway involves an “ATP-induced ATP release” mechanism whereby ATP stimulation of ionotropic P2X or metabotropic P2Y receptors signals intracellular components that favor opening of Panx1 channels (pannexons). ATP is then released from cells. Higher concentration of extracellular ATP released from the cell then acts to close the open pannexon in a negative feed-back loop (Qiu and Dahl, 2009). Linked to paracrine (calcium) wave signaling, this “ATP-induced ATP release” allows for a small, localized number of cells to be stimulated and respond to stresses such as metabolic inhibition, mechanical stress and invading pathogens (Dubyak, 2009). Panx1 is part of the cryopyrin and neuronal inflammasome (Kanneganti et al., 2007; Silverman et al., 2009). The inflammasome is responsible for activation of inflammatory processes and induces pyroptosis, a process of programmed cell death distinct from apoptosis. In particular, Panx1 has been found to co-immunoprecipitate with components of the neuronal inflammasome suggesting that the central nervous system (CNS) inflammasome is pre-formed (Silverman et al., 2009). A recent study showed that Panx1 channels in the hippocampus contributed to seizures by releasing ATP when induced by kainic acid and that deletion of Panx1 or the Panx1 channel blocker reduced the amount of ATP that is released and improves the behavioral manifestation of seizures (Santiago et al., 2011).

**Figure 1:**
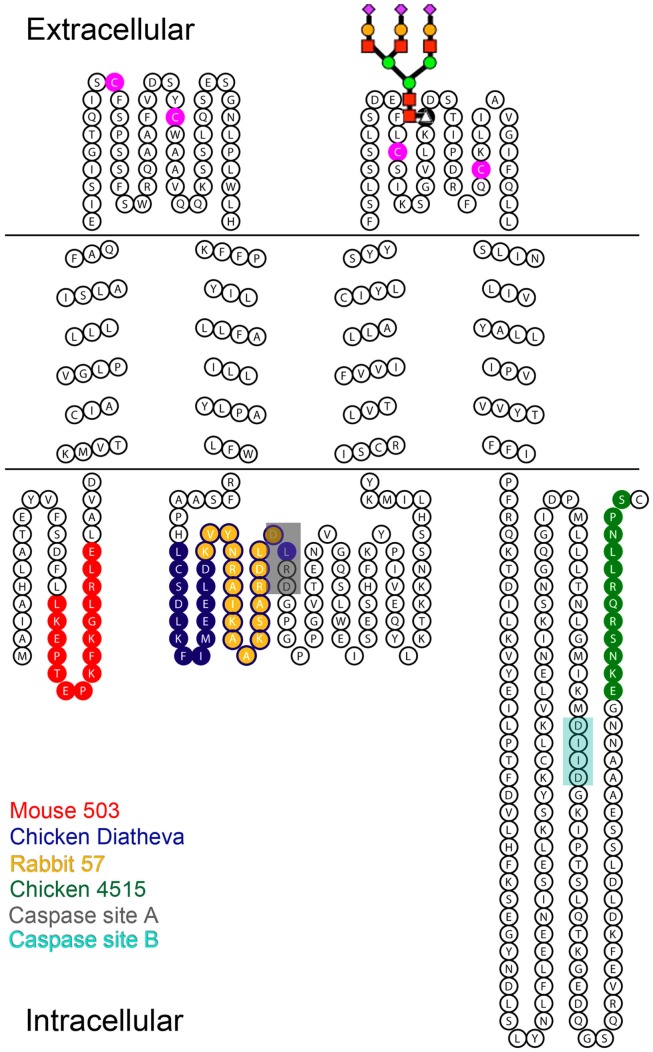
**Diagram of Panx1 topology marked with the epitopes for four different anti-Panx1 antibodies**. Anti-Panx1 antibodies recognize the Panx1 protein at different epitopes (Red – Mouse monoclonal 503 anti-Panx1 (Mo503), Blue – Chicken polyclonal Diatheva ANT0027 (CkDia) anti-Panx1, Yellow – Rabbit polyclonal 57 (Rb57) anti-Panx1, Green – Chicken polyclonal 4515 (Ck4515) anti-Panx1). The extracellular loops of Panx1 contain four cysteine residues (magenta) as well as a glycosylation site at N254. A schematized diagram of the carbohydrate tree is indicated at this position. Caspase cleavage sites are located at residues 164–167 (Chekeni et al., 2010; site A) and 376–379 (Chekeni et al., 2010; site B) and have been shown to be responsive to Casp3 and Casp7.

Immunolabeling studies have shown that Panx1 is widely expressed in cells throughout the body such as CNS neurons, lens epithelial cells, retinal sensory cells, astrocytes, erythrocytes, cardiac myocytes, and macrophages (Dvoriantchikova et al., 2006a; Dvoriantchikova et al., 2006b; Locovei et al., 2006a; Zappala et al., 2006; Zoidl et al., 2007; Karpuk et al., 2011; Kienitz et al., 2011). However, its cellular and subcellular protein distributions in brain regions have not been thoroughly characterized across wide expanses of this complex organ and there have been some conflicting results among different antibodies (Ray et al., 2006; Zappala et al., 2006; Zoidl et al., 2007). In this study, we apply wide field mosaic imaging to examine Panx1 expression in the rat brain across four distinct regions that include cerebellum, hippocampus, and cortex, olfactory bulb, and thalamus while still retaining high spatial resolution. We compared the labeling patterns of four different Panx1 antibodies in rat brain tissue in addition to model cell culture systems using Western blot analysis, and immunocytochemistry. Three of these antibodies are polyclonal antibodies (one raised in rabbit and two in chicken) while the fourth is a mouse monoclonal antibody. Two of these antibodies, one from Diatheva and the other developed and validated in Gerhard Dahl’s laboratory, have been used in several publications (described in more detail in the Results section) while two were developed and validated by our laboratory. The three polyclonal antibodies show fairly similar neuronal labeling patterns across the four brain regions while the Mo503 monoclonal antibody also labeled the same neurons, but with different subcellular localizations. The four antibodies recognize four different epitopes across the protein (Figure 1). In tissue culture cells, we found that both the immunofluorescence and Western blot labeling were highly similar, however there are significant differences in Western blots of tissue among these antibodies.

Because Western blots are frequently treated as “gold standard” controls, especially when used in combination with KO mouse models, there is the widely held opinion that Western blot bands ultimately determine specificity. A recent publication exemplifies this debate by showing that (1) numerous antibodies pass all tests of specificity except the final test in KO mouse tissue, (2) some antibodies pass all tests of specificity by Western blot, but not when using brain tissue, and (3) numerous antibodies pass all rigorous tests of specificity by Western blot, but fail when used for immunohistochemistry (Herkenham et al., 2011), causing the authors to re-assess the results in previous publications that used those antibodies. In the case of pannexins, immunolabeling results have been unclear and controversial. For example, in the study of Panx1 knockout mice by Bargiotas et al. (2011) where *in situ* hybridization images of Panx1 KO brain tissue were devoid of staining for Panx1 transcripts, the authors state that only one antibody (Penuela et al., 2007) out of six tested showed specificity for Panx1 in Western blot of their knockout animals. The Diatheva and Dahl antibodies were two of the five the authors claim to be non-specific. It has always been the case that specificity of antibodies, particularly when used on intact tissues, is hard if not impossible to prove. By these criteria, the results obtained in Western blots in the present study should cause us to discard our findings. However, it should be pointed out that the extra bands we see on Westerns of tissue lysates may represent cellular constituents present at the more complex tissue level that are unfolded on SDS gels. These unfolded proteins may expose amino acid sequences similar to the epitopes used to generate antibodies that are not accessible in intact tissue with folded proteins and not present in simpler cultured cells. This may be especially true since most antibodies these days are generated by short peptides and not folded proteins. We previously showed that connexin antibodies generated against the same C-terminal peptide are highly conformation dependent (Sosinsky et al., 2007).

Here, we advocate for a more balanced approach where we compare all data (Western blots and images from several antibodies) to each other to look for conserved and variable features. We believe that consistency among imaging and cell culture controls with recombinant proteins are also important for antibody validation. Tests of these antibodies on tissue lysates from two different Panx1 KO mice demonstrate that they recognize Panx1 bands, however additional bands are labeled in the KO for some of the antibodies and these vary between KO animals. Thus, it is still unclear whether differences seen between the four antibodies are due to different epitopes being recognized, non-specificity, or the presence of some residual Panx1 protein in KO tissues. Nevertheless, scientific papers continue to be published with these antibodies and/or these KO mouse models, so we discuss strategies for publishing this kind of data by recommending several standards.

## Materials and Methods

### Antibody information

For immunofluorescence experiments, we used either goat anti-Myc (Abcam, Catalog# ab19234, Cambridge, MA, USA) or mouse anti-Myc (Abcam, Catalog# ab32, Cambridge, MA, USA) for co-labeling with either mouse monoclonal anti-Panx1 or rabbit polyclonal anti-Panx1 antibodies, respectively. Both antibodies were used at a 1:250 dilution. GFAP, an astrocytic marker, was labeled using a Guinea pig anti-GFAP (Advanced ImmunoChemical Inc., Long Beach, CA, USA) at a 1:800 dilution.

The four Panx1 antibodies used in this study were (1) a commercial chicken anti-Panx1 (Diatheva ANT0027, denoted as *CkDia*, Diatheva, Fano, Italy; NIF antibody registry number: AB_10013320), (2) chicken anti-Panx1 (obtained from Gerhard Dahl, University of Miami and denoted as *Ck4515*; NIF antibody registry number: AB_10013321), (3) rabbit anti-Panx1 (denoted as *Rb57*; NIF antibody registry number: AB_10013322), and (4) mouse anti-Panx1 (denoted as *Mo503*; NIF antibody registry number: AB_10013323). All antibodies were affinity purified using standard procedures.

(1) *CkDia:* chicken polyclonal antibody CkDia generated against a peptide containing amino acids corresponding to 135–165 of the cytoplasmic loop of the mouse Pannexin1. This antibody was previously characterized in mouse brain by Zappala et al. (2006).
(2) *Ck4515*: chicken polyclonal anti-human Panx1 antibody (#4515, a gift from Dr. Gerhard Dahl at the University of Miami Medical School) raised against a peptide containing the most C-terminal amino acid residues (EKNSRQRLLNPS) in the human Panx1 sequence (Locovei et al., 2006a) and shows cross-reactivity between rat, mouse, and human Panx1 (Boassa et al., 2007).
(3) *Rb57:* rabbit polyclonal antibody Rb57 generated for our laboratory by Abgent, Inc. (San Diego, CA, USA) against a peptide containing amino acids 148–164 (KVYNRAIKAAKSARDLD) of the first intracellular loop. This sequence is invariant among mouse, rat, and human sequences.
(4) *Mo503:* monoclonal antibody Mo503 generated by Abgent, Inc. (San Diego, CA, USA) for our laboratory against a peptide containing amino acids 17–31 (LKEPTEPKFKGLRLE) of the N-terminus of Panx1 and denoted as Mo503. This sequence is invariant among mouse, rat, and human sequences.

The characterization of the Laird laboratory antibody, denoted RbCT-395, is described in Penuela et al. (2007). Its epitope is at residues 395–409 (QRVEFKDLDLSSEAA) of mouse Panx1 and is just upstream of the end of the C-terminus.

### Cell culture and immunocytochemistry

HeLa cells (ATCC Catalog #CCL-2, Manassas, VA, USA) were cultured in Cellgro DMEM (Mediatech, Inc., Manassas, VA, USA) medium supplemented with 10% FBS in a 37°C incubator with 10% CO_2_. Transient transfections were carried out using 2 mL of Lipofectamine reagent (Invitrogen, Carlsbad, CA, USA) and 0.5 mg of DNA encoding Panx1-myc fusion protein in a pRK5 plasmid (Bruzzone et al., 2003). This transfection mixture was added into each cell culture well containing 2 mL of medium and allowed to incubate with cells for 2–4 h. The transfection medium was removed and replaced with regular growth medium. Transfected cells were allowed to continue growing for 24–48 h after transfection then fixed and prepared for fluorescent imaging. Coverslips with transfected HeLa cells were fixed in 4% paraformaldehyde for 20 min at room temperature (*CkDia* and *Ck4515*) or fixed with ice cold 100% methanol for 10 min then rinsed into PBS to rehydrate (*Rb57* and *Mo503*). The different fixation methods were used because while the CkDia, Ck4515, and Mo503 nicely label Panx1 in tissue culture cells and brain tissue using 2–4% paraformaldehyde, in these dual labeling experiments, the goat anti-Myc antibodies gave significant background with paraformaldehyde fixation. For these experiments, anti-Panx1 antibodies were used at 1:250 dilutions with an overnight incubation at 4°C, and all secondary antibodies were used at 1:100 for 1–2 h at room temperature. The cells were briefly permeabilized with 0.1% Triton X100, then blocked with 1% BSA, 3% normal donkey serum, 50 mM glycine, and cold water fish gelatin in PBS. A summary of conditions for each of these antibodies is shown in Table 1. For fluorescence visualization, FITC and Cy5 conjugated donkey secondary antibodies were used (Jackson ImmunoResearch Laboratories, Inc., West Grove, PA, USA). Confocal images stacks were obtained using an Olympus Fluoview 1000 microscope (Olympus USA, PA, USA).

**Table 1:** **Summary of labeling information for the four Panx1 antibodies used in this analysis**.

Panx1 antibody	Source	Catalog #	Antibody ID#	Cell fixation	Tissue fixation	Antigen retrieval
CkDia	Diatheva	ANT0027	AB_10013320	2% PFA or MeOH	4% PFA	No
Ck4515	Laboratory of G. Dahl		AB_10013321	2% PFA or MeOH	4% PFA	No
Rb57	This lab		AB_10013322	MeOH	4% PFA	Yes
Mo503	This lab		AB_10013323	2% PFA or MeOH	4% PFA	No

*Various conditions were utilized to achieve performance with each antibody in the applications presented in this body of work. PFA, paraformaldehyde; MeOH, cold methanol. Use this table to quickly reference these differences in specimen preparation with the unique NIF antibody ID (Neuroscience Information Framework Antibody Identifier http://antibodyregistry.org/antibody17/antibodyform.html?gui_type = advanced)*.

### Preparation of rat brain tissue and immunohistochemistry

All experiments involving vertebrate animals conform to the National Institutes of Health *Guide* *for the Care and Use of Laboratory Animals* and were approved by the Institutional Animal Care and Use Committee of the University of California San Diego. The animal welfare assurance number is A3033-01. Rats were fully anesthetized and Ringer’s solution containing xylocaine and heparin was perfused transcardially for 3 min followed by 4% paraformaldehyde for 10 min. Here, we show images obtained from an 8-week-old female Sprague-Dawley, however, mosaic images from other rat brains we examined had the same Panx1 patterns. The brain was removed and post-fixed in 4% paraformaldehyde overnight at 4°C. Sagittal sections were cut on a Leica vibratome at a thickness of 75 microns and stored at −20°C in cryoprotectant solution (30% glycerol, 30% ethylene glycol in PBS) until processed.

Before immunolabeling, each sagittal section was severed into the four regions to be imaged (cerebellum, thalamus, olfactory bulb, and hippocampus) to facilitate optimal flatness when mounting on slides for subsequent wide scale imaging. Tissue sections to be labeled with the Rb57 anti-Panx1 antibody underwent antigen retrieval using heat and 10 mM citric acid pH 6.5 for 10 min (100°C water bath in microwave oven; von Wasielewski et al., 1994). Free-floating tissue sections were blocked with 3% normal donkey serum, 1% bovine serum albumin, 1% cold water fish gelatin, 0.1% Triton X100, and 50 mM glycine in PBS for 1 h at room temperature. Guinea pig anti-GFAP and anti-Panx1 primary antibodies were applied overnight at 4°C. FITC and Cy5 or FITC and Rhodamine RedX conjugated donkey secondary antibodies (Jackson ImmunoResearch Laboratories, Inc., West Grove, PA, USA) were applied for 2.5 h at room temperature. The Mo503 primary antibody was detected using a Rhodamine RedX conjugated donkey anti-mouse secondary antibody having minimal cross-reaction to rat in order to avoid detection of endogenous rat immunoglobulin in the tissue (Jackson ImmunoResearch Laboratories, Inc., Catalog# 715-295-151). The immunolabeled tissue samples were incubated with 0.5 μM DAPI nuclear stain (Invitrogen, Carlsbad, CA, USA) for 20 min at room temperature and carefully mounted as flat as possible using gelvatol as the mounting medium.

### Western blots

Lysates were prepared from MDCK cells stably over-expressing Panx1 in addition to endogenous Panx1 as previously described (Boassa et al., 2007; Ambrosi et al., 2010). Normal rodent brain lysates were obtained from G-Biosciences (Maryland Heights, MO; GenLysate Protein lysates of normal rat brain NLR-02, and normal mouse brain NLM-02). Normal rodent brain lysates were also obtained from Enzo Life Sciences, Inc. (Farmingdale, NY, USA). These included mouse brain lysates, Immunoblotting Standard SW-104, and rat brain lysates, Immunoblotting Standard SW-103. Proteins in the lysates were separated by SDS-PAGE on a 4–20% pre-cast gel (Invitrogen, Carlsbad, CA, USA). Proteins were transferred to PVDF membrane (Millipore, Billerica, MA, USA), using NuPAGE transfer buffer (Invitrogen). Bands on Western blots were identified using the four anti-Panx1 antibodies with HRP-conjugated secondary antibodies (Calbiochem); the chemiluminescence reaction was visualized using SuperSignal West Pico ECL (Thermo Scientific, Waltham, MA, USA) and Kodak Gel Logic 2200 gel visualizer (Carestream Health, Inc., Rochester, NY, USA) or X-ray film. The Mo503 and Rb57 antibodies were used at a 1:5000 dilution in blocking solution (1X PBS-T with 5% milk). The Ck4515 and CkDia antibodies were diluted 1:2000 in a blocking solution made with 2% milk and 0.5 M NaCl in 1X PBS-T. This stringent condition significantly reduced the background of these two antibodies.

Lysates from matched Panx1 wild type and KO tissues were generated from animals from Genentech (Qu et al., 2011) and KOMP (Qiu et al., 2011; Santiago et al., 2011). Methods for the preparation of tissues lysates and Western blots for the KOMP KO are described in (Santiago et al., 2011). General methods for preparation of tissue lysates from the Genentech animals were prepared using methods as described in Penuela et al. (2007).

### Wide scale fluorescent imaging

Overlapping 3D image stacks were acquired with an Olympus FluoView 1000 laser scanning confocal microscope equipped with a 40×, NA 1.3, oil-immersion objective lens, and a special *x*, *y*, *z* montaging stage. A multi-area time-lapse was performed using the Olympus ASW1.7 software to define the boundaries of the tissue area for the acquisition volume. The individual tiles of the montage were acquired as *z* stacks of 4–6 sections (∼0.5–0.7 mm/section) using sequential line scanning and then a maximum intensity projection of each stack is computed to make the individual tiles. The tiles were stitched together post-data acquisition using National Center for Microscopy and Imaging Research (NCMIR) developed ImageJ plugins to project, flat field, normalize, align, and combine into a mosaic (Chow et al., 2006; Kenyon et al., 2010). Software is available for download^1^. The resulting reconstructed mosaic image was downloaded and opened in Photoshop (Adobe Systems Inc., San Jose, CA, USA) to rotate, crop, and adjust color balance of the image.

### Image deposition into the cell centered database

Mosaic images were acquired using an automated imaging workflow system developed at NCMIR to upload imaging data with its associated metadata directly from the microscopes in our facility and register them as microscopy products within the CCDB (Bouwer et al., 2011). This complex system for data collection, storage, and manipulation funnels large numbers of valuable datasets directly into the CCDB (Martone et al., 2008). Eighteen datasets resulting from this publication have been released to the public through the CCDB. Our large scale mosaics can be browsed or downloaded through the Cell Centered Database (CCDB; Martone et al., 2002, 2008) at http://ccdb.ucsd.edu by selecting project ID P20002. Because these data sets are quite large and rich in information, each of these reconstructed mosaic images (usually 1–4 GB in size) is viewable at full resolution and annotatable without downloading using the Web Image Browser (WIB^2^), a visualization program based on GIS technology similar to that used for Google maps, but specialized for microscopy imaging data. Through the WIB, users can turn on and off channels and adjust contrast of large microscopic imaging data sets.

## Results

This work focuses on the characterization of four different anti-Panx1 antibodies in selected areas of the rat brain and compares cellular localizations. In our study, we ask the question: “What are the similarities and differences in tissue labeling patterns between four antibodies generated against different parts of the same molecule?” The diagram in Figure 1 shows the topology of the Panx1 monomer and the epitopes of the four anti-Panx1 antibodies examined. Common features provide clues as to how Panx1 is localized to cellular sub-types, while dissimilar labeling may provide information about possible differences between neurons or microenvironments in one area of the brain versus another. It is difficult to point to which antibody is specific or non-specific as the Rb57 antibody produces the most bands in a Western blot, however the overall pattern of labeled cells in brain tissue matches that of the Ck4515 and CkDia with the exception of cells lining the vessels that are not labeled by Ck4515. As noted previously, epitopes are highly conformation dependent and accessibilities may be different in tissues where proteins are folded as opposed to Western blots were proteins are unfolded. Incubation of the peptide used for generating Rb57 antibody eliminated immunofluorescence staining in canine cardiac tissue (Dolmatova et al., 2012). Differences in the labeling patterns may also indicate non-specific interactions of the antibody that can occur due to recognition of similar short amino acid sequences. For Panx1, this has been especially problematic (Bargiotas et al., 2011) indicating unanticipated complexity when comparing simple tissue culture systems with intact tissue. Two of these antibodies have already been characterized in publications (CkDia and Ck4515), while the other two were developed by us using a contracted company and validated within our own laboratory. The specificity of the Ck4515 anti-Panx1 antibody was characterized in a previous publication by Western blot and immunofluorescence staining in oocytes, erythrocytes and heart capillaries (Locovei et al., 2006a). While not explicitly stated in Locovei et al. (2006a), the Ck4515 antibody was validated for specificity in exogenously expressing oocytes using peptide competition experiments and incubation with preimmune serum (Gerhard Dahl, personal communication). This antibody was reported to show no immunofluorescence in staining of Panx1 KO mouse tissue (Zoidl et al., 2007) and has been used with several tissues (Ransford et al., 2009; Silverman et al., 2009; Dolmatova et al., 2012). The specificity of CkDia antibody was originally characterized in mouse brain and in transfect cells by Zappala et al. (2006) who demonstrated a single band on Western blot that was absent in parental HeLa cell lysate and eliminated by preabsorbing the antibody with the immunizing antigen. CkDia has been used in several publications (Romanov et al., 2007; Tang et al., 2008; Zhang et al., 2008; Huang et al., 2011).

However, none of these antibodies has been compared to each other using the same specimens and controls. As part of these controls, we present Western blot data on Panx1 KO mice tissues. It should be noted that while Panx1 labeling with recombinant Panx1 in cultured cells gave consistent results, the Western blots from KO animals were not entirely clean or consistent between tissues and/or KO mice.

### Panx1 labeling in cultured cells

**Figure 2:**
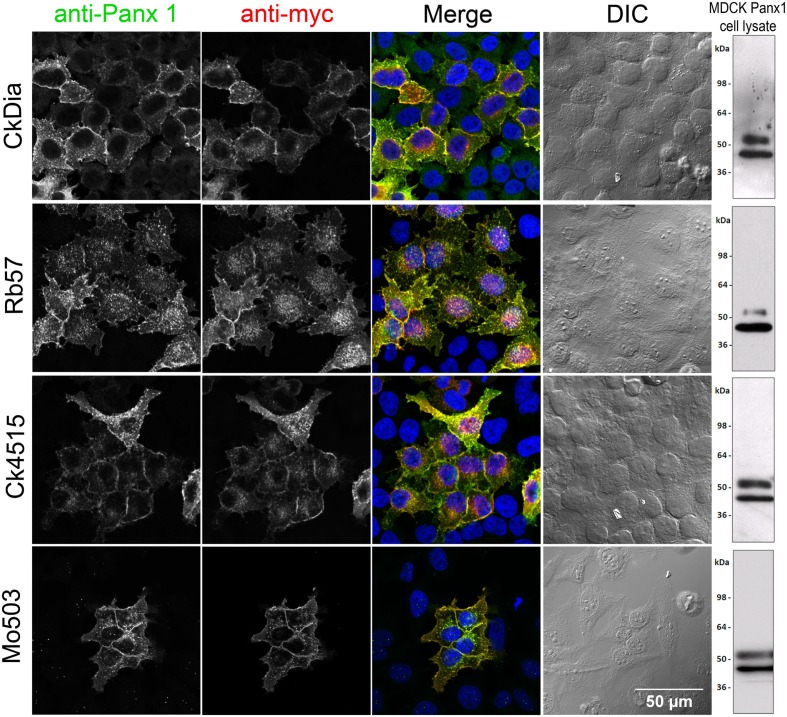
**Anti-Panx1 and anti-myc antibodies recognize Panx1-myc transiently expressed in HeLa cells**. From left to right, each row of images shows anti-Panx1, anti-myc, and a color overlay of the previous two images with Panx1 in green, myc in red, and DAPI in blue, followed by the corresponding DIC image. Note the overlap of the Panx1 and myc staining and the lack of signal in untransfected cells. Each of these antibodies against Panx1 decorates the plasma membrane as well as intracellular localization. A Western blot for Panx1 in cell lysates prepared from over-expressed Panx1 stably expressed in MDCK cells for each of these antibodies is shown in the right hand column.

Each of these four antibodies was first tested using transfected Panx1-myc HeLa cells, a system where there is no endogenous Panx1, to ensure that the antibody labeled exogenously expressed tagged Panx1 (e.g., myc, Flag, His etc.), and showed almost complete overlap with an antibody for the epitope tag. For immunofluorescence experiments, HeLa cells transiently transfected with Panx1-myc demonstrated the specificity of each of the four anti-Panx1 antibodies based on the overlap of Panx1 with commercial and well-characterized myc antibodies (Figure 2). A negative control omitting primary antibodies from the initial incubation showed no labeling (unpublished results). In addition, no staining corresponding to the glycosylated or unglycosylated Panx1 bands were obtained in Western blots of parental HeLa cell lysates for these four antibodies (data not shown; Zappala et al., 2006). These four anti-Panx1 antibodies performed similarly in labeling cultured cells. Figure 2 shows that in non-expressing cells there is typically little or no fluorescence. Co-localization of the labeling pattern between Panx1 protein (grayscale, column1) and the myc tag (grayscale, column2) resulted in yellow in the merged RGB image (column 3) that showed expression at the cell membrane as well as in intracellular populations, as has been previously published (Boassa et al., 2007; Penuela et al., 2007; Boassa et al., 2008; Penuela et al., 2009). The CkDia Panx1 antibody displayed some low-level background fluorescence in non-transfected cells when compared to the myc labeling, that is not present with the other three antibodies. These antibodies were also tested for immunofluorescence with transfected HEK 293T cells, and MDCK cells over-expressing Panx1 and showed the same overlapping labeling patterns (unpublished results).

**Figure 3:**
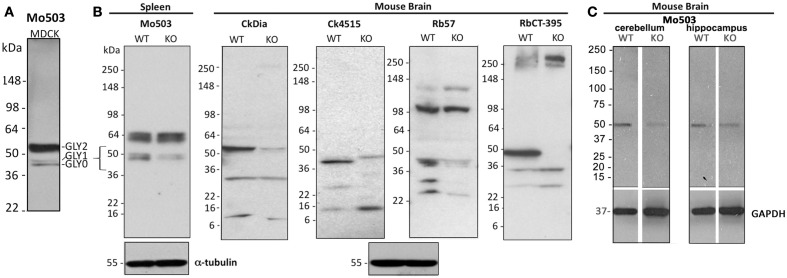
**Western blot analysis of Panx1 protein in cell and brain lysates probed with four different anti-Panx1 antibodies**. **(A)** Banding pattern of MDCK lysates at the far left, glycosylation bands are noted as: GLY0, GLY1, GLY2. **(B)** Tissue lysates from either brain or spleen from the Genentech generated KO mouse are examined by Western blot with the four antibodies used for imaging studies. The bracket at left highlights the ∼43–55 kDa region where bands for Panx1 species are expected and show decreases in the KO tissue. The Western blot at the far right uses the CT-395 antibody developed by the Laird laboratory and serves as a control for matching against our antibodies. α-tubulin blots are shown as a loading control. **(C)** Cerebellum and hippocampus tissue lysates from a KOMP generated Panx1 KO show decreased intensity from wild type in Western blots. Here, Western blotting against GAPDH was used as a loading control.

We used over-expressing Panx1 MDCK cells containing both endogenous Panx1 and exogenous untagged Panx1 for our Western blot analysis (Figure 2 *right hand column*). These cells provided a very strong signal for detecting Panx1 in Western blots and have two strong bands ∼48 and ∼52 kDa in size that are labeled by all four antibodies. It has been previously established that Panx1 in SDS-PAGE/Western Blots runs as three bands that are correlated with their glycosylation status (Boassa et al., 2007; Penuela et al., 2007). The Western blot in Figure 3 far left panel shows that the lower band in Figure 2 was resolved into two bands using a different lysate of the same cell line. This pattern of three bands was evident with each of the four antibodies tested (unpublished results). We have previously named these three bands as GLY0 (non-glycosylated, bottom band), GLY1 (partially glycosylated, middle band), and GLY2 (fully glycosylated, upper band). Our previous analyses (Boassa et al., 2007, 2008) and that of others (Penuela et al., 2007) demonstrated that the fully glycosylated GLY2 Panx1 species localized to the plasma membrane while the GLY0 and GLY1 Panx1 species are found in intracellular compartments. It should be noted that parental MDCK cells containing only endogenous Panx1 show a similar profile, but the GLY0, GLY1, and GLY2 bands have weaker intensities consistent with less expression (Penuela et al., 2007). These Panx1 protein bands were used to compare for molecular weights with endogenous Panx1 found in brain tissue (Figure 3).

### Western blot labeling of brain tissue

While model cultured cells represent excellent homogeneous and easily manipulated systems for understanding expression and trafficking, we focused our study on expression patterns of Panx1 in the more complex organization of brain tissue, a heterogeneous organ containing several cell types intermingled and interacting within spatial domains. We originally tested Western blots of commercially available rat and mouse brain lysates with all four antibodies (data not shown). We analyzed the rodent brain lysates after SDS-PAGE and Western blotting and the Panx1 banding patterns comparing rat to mouse brain were similar. Although each of the four antibodies gave highly similar banding patterns in tissue culture cells, there were variations in the banding patterns in Westerns of the same rat and mouse brain lysates using these four Panx1 antibodies. We also performed Western blots of rat cerebellum lysates, however the banding pattern for each antibody looked the same as the total rat brain lysates.

As additional controls for validating our antibodies using Western blots, we analyzed banding patterns between wild type and KO mice generated independently by two different labs (Qu et al., 2011; Santiago et al., 2011). Contrary to what has been reported by Bargiotas et al. (2011), we see a reduction or elimination of bands in the 36–55 kDa region of expected Panx1 bands (see bracketed region indicated in the far left Western) of the KO tissue with all antibodies. However, we also saw bands in the ∼20 kDa range that also were eliminated or decreased. Residual weak bands in the KO lanes may be a result of incomplete knock-down of the Panx1 protein in these two knockout models. Every antibody tested, even CT-395 (which Bargiotas et al. stated was the one successful antibody to show knockout of Panx1), showed additional bands that are not eliminated or reduced in a KO tissue. As an example, the Mo503 antibody showed a much more dramatic reduction in spleen (Figure 3B) than in brain KO tissues (Figure 3C). Additionally, the 64 kDa bands in the spleen lysate of the Genentech KO mouse were not present in the brain lysates of the KOMP KO mouse. Particularly, while the CT-395 antibody Western blot in Figure 3 does not show Panx1 bands in the knockout lane in this figure, longer exposures of the Western blot show Panx1 bands in the expected 36–55 kDa region (data not shown). Thus, we not only saw differences between different antibodies, but also differences between KO models and even KO tissues from the same animals. It is important to note that a quantitative real time polymerase chain reaction (qRT-PCR) analysis of Panx1 transcripts in several tissues demonstrated that Panx1 knockout animals generated by KOMP using the knockout first strategy (Testa et al., 2004) are hypomorphs rather than true knockouts (Hanstein et al., submitted).

### Panx1 labeling in four brain regions

**Figure 4:**
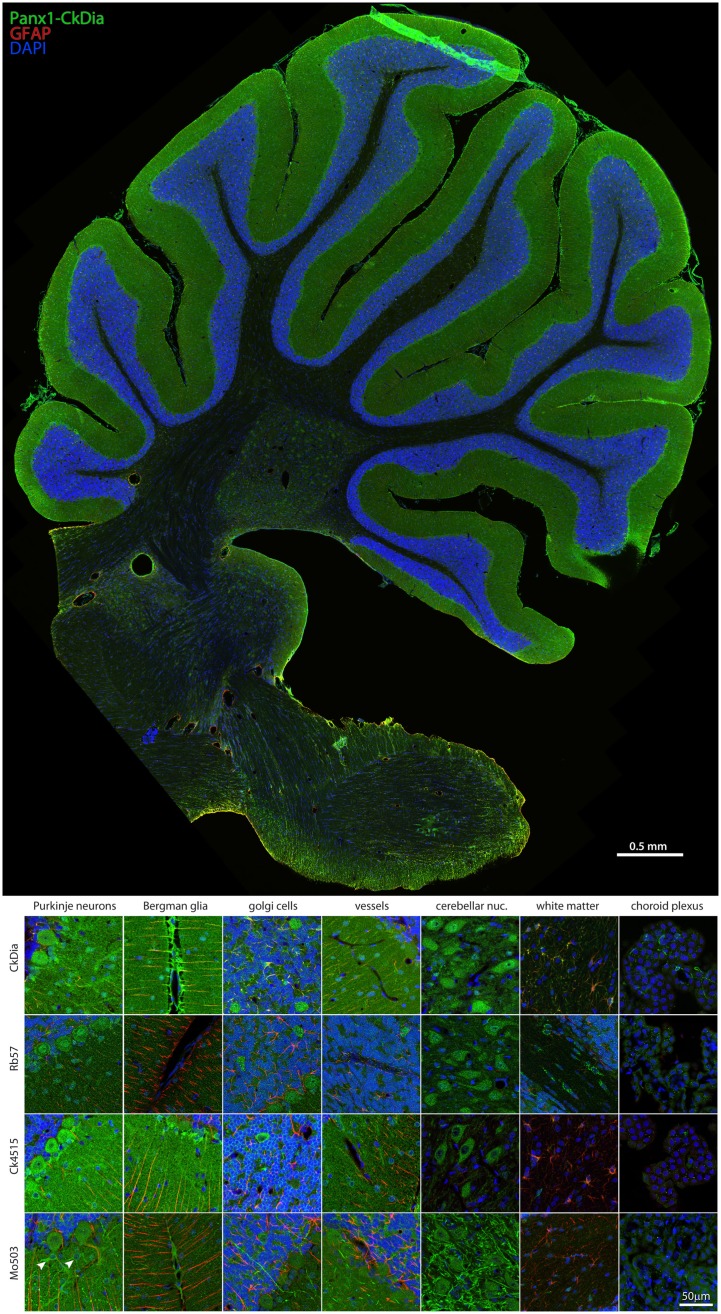
**Large scale mosaic imaging of rat brain cerebellum**. Top: this representative cerebellum montage is labeled with CkDia anti-Panx1 antibody (green), GFAP (red), and DAPI (blue, nuclei). This mosaic image is made up of 587 tiles. Each tile is a maximum intensity projection of a stack of five Z-sections that were stitched together to reconstruct this single, high-resolution 2D image. Bottom: full resolution views of cerebellar regions and cell types labeled by the four anti-Panx1 antibodies. Arrowheads in the Mo503 left hand image = stellate cells.

**Figure 5:**
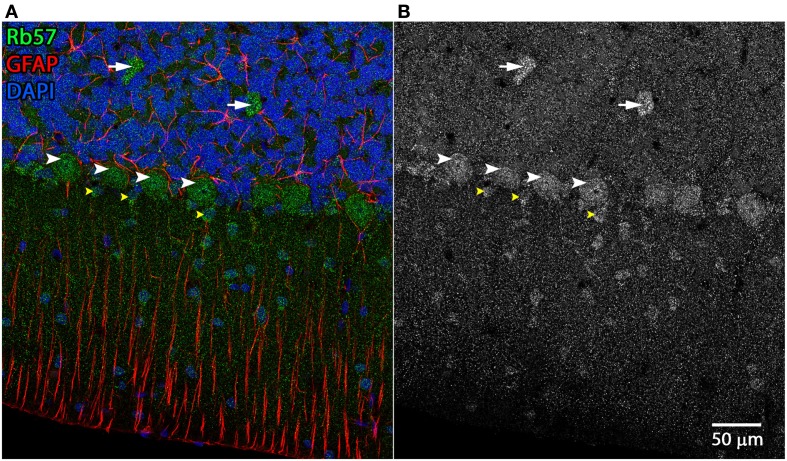
**Cerebellar cell types labeled by Rb57 anti-Panx1 antibody**. Labeling of rat cerebellum tissue with the Rb57 anti-Panx1 antibody reveals expression in Purkinje neurons (white arrowheads), Golgi cells within the granular layer (white arrows), Bergmann glia within the molecular layer of the cerebellum (yellow arrowheads), and additional cell bodies in the molecular layer that may be stellate cells. **(A)** Merged image **(B)** Panx1 channel shown in grayscale.

**Figure 6:**
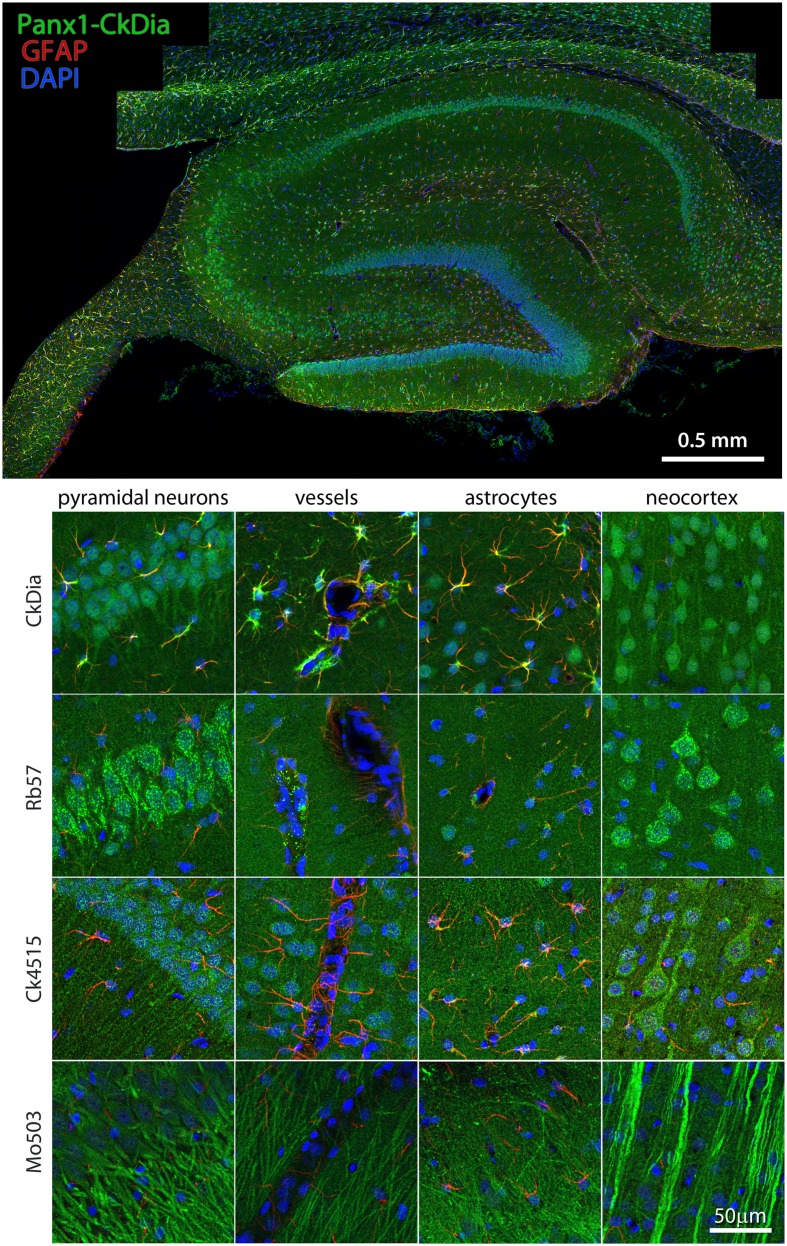
**Large scale mosaic imaging of hippocampus and neocortex lying adjacent to the hipps ocampus**. Top: this Representative hippocampus montage is labeled with CkDia anti-Panx1 antibody (green), GFAP (red), and counterstained nuclei with DAPI (blue). This mosaic image is made up of 167 tiles. Each tile is a maximum intensity projection of a stack of four Z-sections that were stitched together to reconstruct this single, high-resolution 2D image. Bottom: full resolution views of hippocampal regions and cell types labeled by the four anti-Panx1 antibodies.

**Figure 7:**
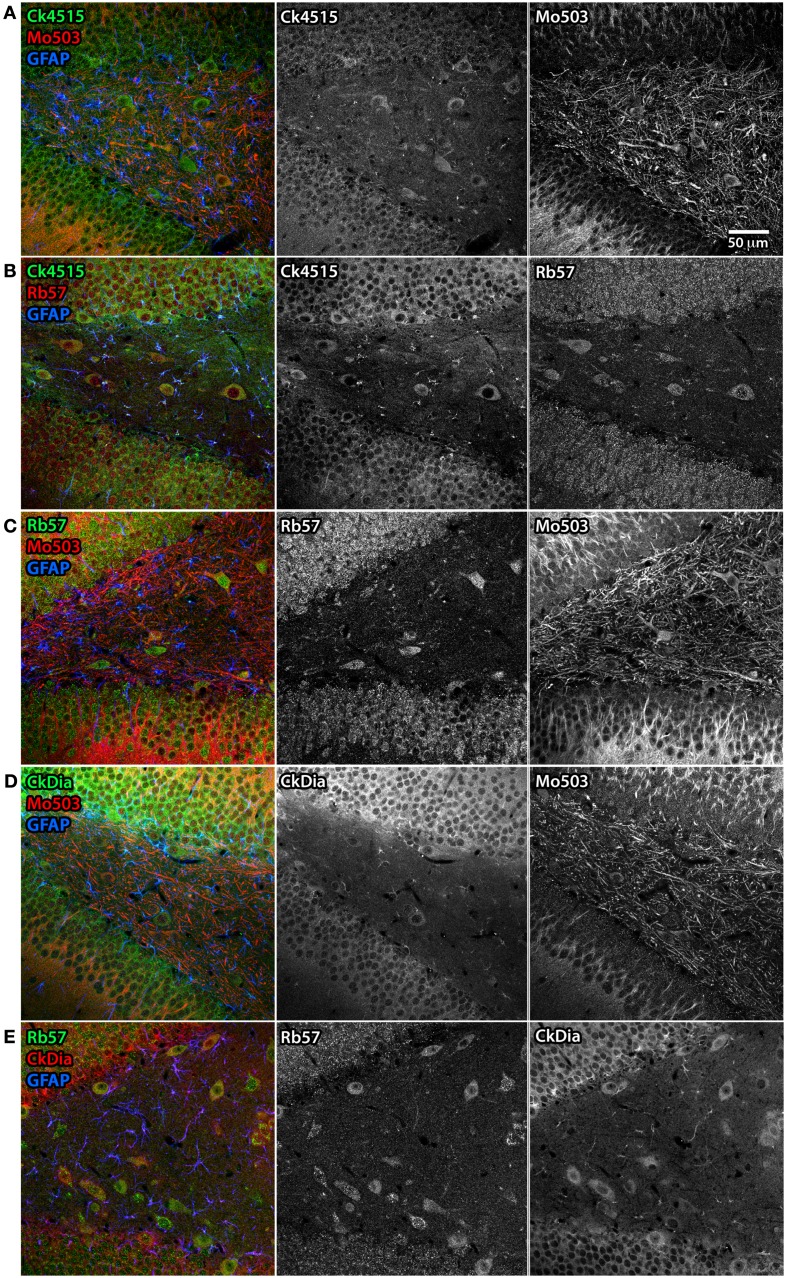
**Differential subcellular labeling of Panx1 in the dendate gyrus of the rat hippocampus with combinations of four anti-Panx1 antibodies**. Although the neurons of the hippocampus are a consensus cell type labeled by all four of the Panx1 antibodies, there are differences in the apparent subcellular localization of the protein population recognized by each antibody. In these five panels, each antibody is shown in black and white (middle and right hand images) and as a composite color image in the left hand images. In all composite images, astrocytes are labeled with anti-GFAP (blue). **(A)** Co-labeling with Ck4515 and Mo503. **(B)** Co-labeling with Ck4515 and Rb57. **(C)** Co-labeling with Rb57 and Mo503. **(D)** Co-labeling with CkDia and Mo503. **(E)** Co-labeling with Rb57 and CkDia. Tissue slices labeled with Rb57 **(B,C,E)** underwent antigen retrieval prior to immunolabeling. Co-labeling tissue with the polyclonal antibodies reveals that these primarily highlight the cell body and the adjacent portion of the dendrite however the Mo503 antibody (red) decorates the long dendrites of these cells with less noticeable staining at the cell bodies. However, there is a substantial degree of overlap in staining between all the antibodies.

**Figure 8:**
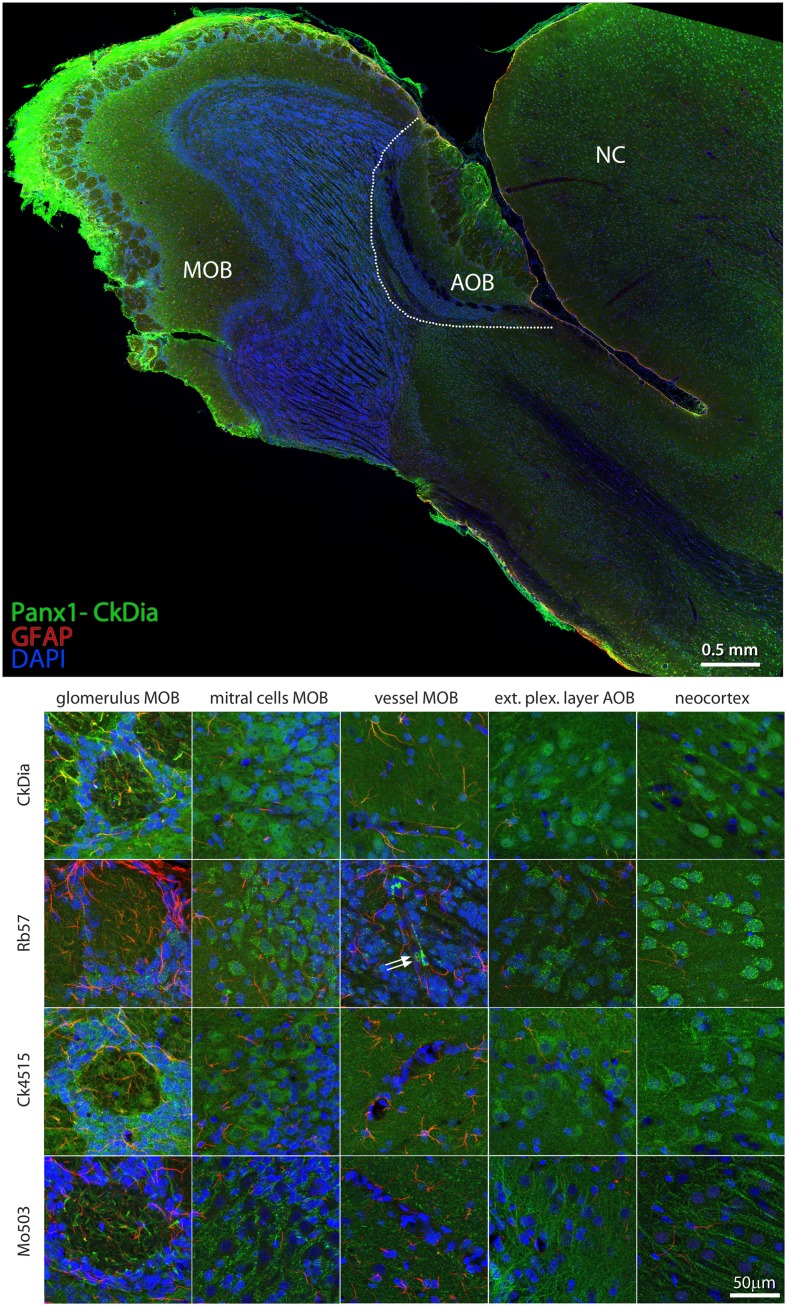
**Large scale mosaic imaging of rat brain olfactory bulb and neocortex lying adjacent to the olfactory bulb**. Top: representative montage labeled with CkDia anti-Panx1 antibody (green), anti-GFAP (red), and DAPI (blue, nuclei). This mosaic image is made up of 649 tiles. Each tile is a maximum intensity projection of a stack of five Z-sections that were stitched together to reconstruct this single, high-resolution 2D image. Bottom: full resolution views of olfactory bulb regions and cell types labeled by the four anti-Panx1 Abs. White arrows = red blood cells, MOB, main olfactory bulb; AOB, accessory olfactory bulb; NC, neocortex.

**Figure 9:**
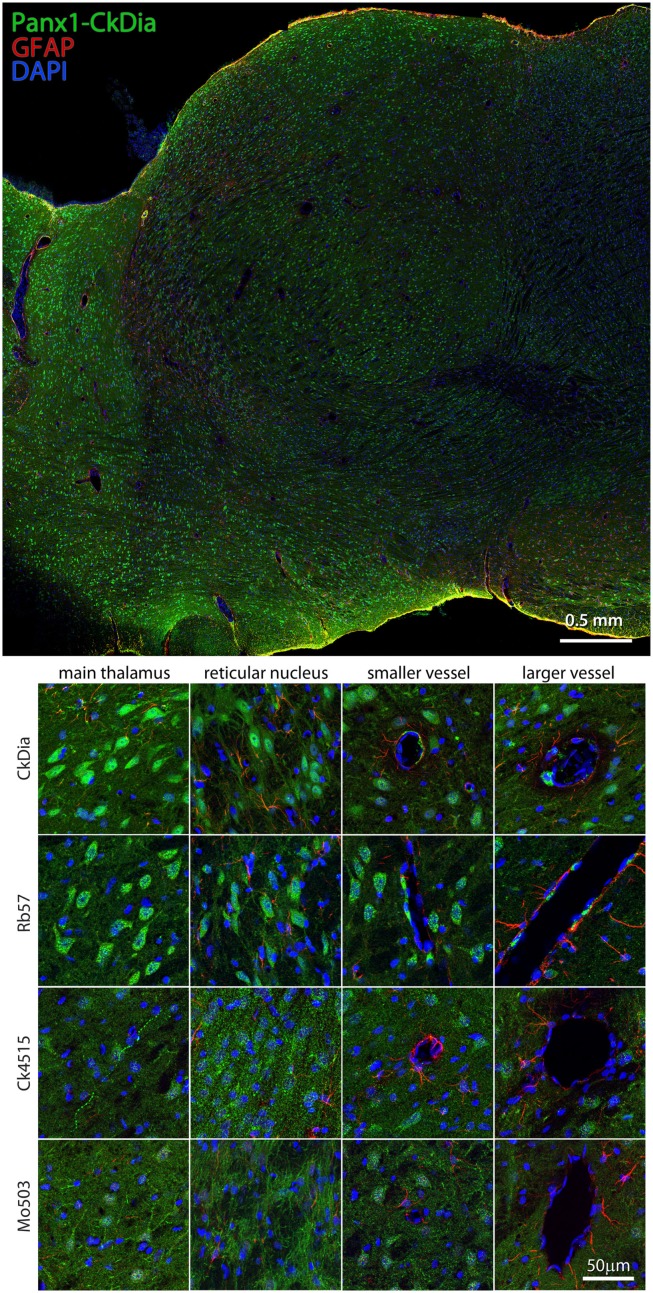
**Large scale mosaic imaging of rat brain thalamus**. Top: representative thalamus montage labeled with CkDia anti-Panx1 (green), anti-GFAP (red), and DAPI (blue, nucleus). This mosaic image is made up of 447 tiles. Each tile is a maximum intensity projection of a stack of five Z-sections that were stitched together to reconstruct this single, high-resolution 2D image. Bottom: full resolution views of thalamus regions labeled by the four anti-Panx1 antibodies.

We focused on cerebellum, hippocampus, olfactory bulb, and thalamus, four areas of the rat brain that have all been reported to have high Panx1 expression using *in situ* hybridization in either mouse or brain tissue (Ray et al., 2005; Vogt et al., 2005; Weickert et al., 2005; Lein et al., 2007). Wide scale immunofluorescence mosaic images allowed us to image large morphological domains while still retaining labeling information at the cellular resolution scale. Immunolabeled sagittal rat brain slices corresponding approximately to a lateral position of 1.40–2.10 mm of the Paxinos and Watson (1998) rat brain atlas were imaged in four large regions (Figures 4–9). In addition, the hippocampus and olfactory bulb images contain areas of nearby neocortex. In Figures 4, 6, 7, and 9, we show only one montage using the CkDia antibody but also show full resolution views from within montages of brain regions labeled with each of the four antibodies. The Panx1 labeling is always shown in green. Each tissue slice was also labeled with anti-GFAP antibodies to distinguish astrocytes (red) while cell nuclei were stained with DAPI (blue).

Because image data is rich in information and each montage at full resolution is typically over 2 GB, we chose to make all montages publicly available, rather than show down-sampled mosaics, except as an example for each area. Each of these mosaics at full resolution has been deposited in the CCDB under project ID P20002 and can be viewed using the Web Image Browser (WIB) or downloaded onto a reader’s computer via the CCDB portal^3^. Table 2 contains URLs linking to each of these large data sets for viewing using the WIB.

**Table 2:** **List of URLs linking to display of full resolution large-scale mosaic images using four different anti-Panx1 antibodies of four regions within the rat brain studies here**.

Brain region	Panx1 Ab	CCDB MP ID	URL
Cerebellum	CkDia	56630	http://purl.oclc.org/NET/56630_CkDia_Cer
Cerebellum (Alternate)	CkDia	57185	http://purl.oclc.org/NET/57185_CkDia_Cer
Thalamus	CkDia	48338	http://purl.oclc.org/NET/48338_CkDia_Thal
Thalamus (Alternate)	CkDia	46648	http://purl.oclc.org/NET/46648_CkDia_Thal
Hippocampus	CkDia	48367	http://purl.oclc.org/NET/48367_CkDia_Hip
Olfactory bulb	CkDia	58640	http://purl.oclc.org/NET/58640_CkDia_Olf
Cerebellum	Ck4515	44959	http://purl.oclc.org/NET/44959_Ck4515_Cer
Thalamus	Ck4515	48920	http://purl.oclc.org/NET/48920_Ck4515_Thal
Hippocampus	Ck4515	48902	http://purl.oclc.org/NET/48902_Ck4515_Hip
Olfactory bulb	Ck4515	48664	http://purl.oclc.org/NET/48664_Ck4515_Olf
Cerebellum	Mo503	45305	http://purl.oclc.org/NET/45305_Mo503_Cer
Thalamus	Mo503	58721	http://purl.oclc.org/NET/58721_Mo503_Thal
Hippocampus	Mo503	58445	http://purl.oclc.org/NET/58445_Mo503_Hip
Olfactory bulb	Mo503	58675	http://purl.oclc.org/NET/58675_Mo503_Olf
Cerebellum	Rb57	45276	http://purl.oclc.org/NET/45276_Rb57_Cer
Thalamus	Rb57	59247	http://purl.oclc.org/NET/59247_Rb57_Thal
Hippocampus	Rb57	56258	http://purl.oclc.org/NET/56258_Rb57_Hip
Olfactory bulb	Rb57	58377	http://purl.oclc.org/NET/58377_Rb57_Olf

*Eighteen full resolution montage images are viewable using the WIB tool by clicking on these URLs. Two high quality montages for which individual images are not shown in the galleries in Figures 4–9 are also available for viewing and downloading and are denoted by “Alternate” under the brain region category. CkDia, Chicken Diatheva ANT0027 polyclonal antibody; Ck4515, Chicken 4515 polyclonal antibody (Dahl laboratory); Rb57, Rabbit 57 polyclonal antibody (Sosinsky lab); Mouse monoclonal 503 polyclonal antibody (Sosinsky lab). Each brain region is displayed with anterior facing left, and the reconstructed images have been cropped and color balance adjusted using Photoshop. All images (raw and processed) as well as associated metadata can be downloaded through the CCDB using the listed microscopy product identification number (MPID)*.

#### Cerebellum

Examples of the cerebellar cell types labeled by the anti-Panx1 antibodies are shown in Figures 4 and 5. These include Purkinje neurons, Golgi neurons, Bergman glia, and their parent cells, the Golgi epithelial cells, as well as cells within the deep cerebellar nuclei, all of which have been previously reported to contain significant amounts of Panx1 in mouse brain (Ray et al., 2006; Zappala et al., 2006). In comparing the staining patterns of these four antibodies (Figure 4, bottom), some patterns of differential antibody labeling across cerebellum tissue became apparent. For example, we found that the Mo503 antibody labels stellate cells or basket cells of the molecular layer (Figure 4, white arrowheads). The Rb57 antibody labeled cell bodies within the white matter while the CkDia antibody labels astrocytes within the white matter (Figure 4, bottom column 6). The three polyclonal antibodies labeled the Bergman glia; however no glial staining was observed with the Mo503 anti-Panx1 antibody (Figure 4, bottom, column 2). In Purkinje neurons, the labeling of all four antibodies was localized to the cell bodies, however, some staining of the axonal processes were visible, especially with the Mo503 antibody. The choroid plexus subcellular distribution was more speckled although some hints of apparent plasma membrane staining can be seen using the CkDia antibody. In the higher magnification view shown in Figure 5, labeling of rat cerebellum tissue with the Rb57 anti-Panx1 antibody revealed expression in Purkinje neurons (white arrowheads), Bergmann glia within the molecular layer of the cerebellum (yellow arrowheads), as well as Golgi neurons within the granular layer (white arrows). In general, all three polyclonal antibodies labeled Panx1 in astrocytes in the cerebellum. This overlap was more obvious when viewing the image through the WIB and the red GFAP channel was turned off.

#### Hippocampus

Examples of hippocampal cell types (and neocortex lying superficial to the hippocampus) labeled by the anti-Panx1 antibodies are shown in Figures 6 and 7. These images contain pyramidal neurons and astrocytes that have been previously reported to express Panx1 at the protein level in mouse brain (Zappala et al., 2006; Zappala et al., 2007; Karpuk et al., 2011). Vessel labeling by CkDia and Rb57 antibodies was difficult to distinguish from astrocyte end feet that are wrapped around the vessels and highly labeled in this brain region. Interestingly, even in instances where all four antibodies label the same cell type in consensus, like in the pyramidal neurons of the hippocampus (Figure 6), there were still differences at the subcellular level. In this case, the three polyclonal anti-Panx1 antibodies label the cell body and adjacent portion of the apical dendrite, while the Mo503 anti-Panx1 labeling is restricted to the dendrites of these cells (Figure 7). In many cell types the Mo503 preferentially labels dendrites or other cellular extensions and weakly labels cell bodies such as in the hippocampal pyramidal neurons and pyramidal cells of the neocortex region, while the polyclonal antibodies often highlight the cell bodies (Figures 4 and 6–9). Astrocytes in this region were labeled with the three polyclonal antibodies, but not by the monoclonal Mo503 antibody.

#### Olfactory Bulb

The anti-Panx1 antibodies shown in Figure 8 labeled several olfactory bulb cell types. These include the mitral cells, cells within the glomeruli of the main olfactory bulb (MOB) and cells within the exterior plexiform layer of the accessory olfactory bulb (AOB), and cells of the adjacent neocortex. In the examples of blood vessels from this region the Rb57 antibody sporadically labels cells lining the vessels, presumptively endothelial cells (Figure 8, bottom, column 3). Panx1 immunostaining has been previously reported in strial blood vessels in the cochlea (Wang et al., 2009) and in the endothelial cells of cardiac capillaries (Locovei et al., 2006a) using the Ck4515 antibody and in endothelial cells of lens capillaries (Dvoriantchikova et al., 2006b) using a different rabbit polyclonal antibody. Two red blood cells that are quite rare with this type of specimen preparation can be seen clearly in this instance within the blood vessel (white arrows), confirming robust Panx1 expression in erythrocytes (Locovei et al., 2006a). Again, as shown in the mitral cells, exterior plexiform layer, and neocortex, the polyclonal antibodies highlighted the neuronal cell bodies much more than the Mo503 antibody that preferentially stained the neuronal processes.

#### Thalamus

Previous *in situ* hybridization studies showed staining with Panx1 mRNA probes in the mouse thalamus and in particular, reticular thalamic neurons (Ray et al., 2005). We found in this tissue, Panx1 localized mostly to neuronal cell bodies, although the Mo503 and Ck4515 do delineate neuronal processes. As with the other brain regions, the Rb57 and CkDia antibodies recognize sporadic cells lining blood vessels, presumably endothelial cells, while the other two do not (Figure 9). Interestingly, this brain region had the least labeling of Panx1 in astrocytes. It has been shown previously that astrocytes cultured from specific brain regions show differential expression patterns of adrenergic receptors (Ernsberger et al., 1990) and the differing astrocytic labeling we see in the various brain regions could be attributed to this phenomenon.

## Discussion

Panx1 is the most studied and best characterized isoform of the pannexin protein family. It has been demonstrated to form large conductance channels in neurons, glial cells, and erythrocytes (Locovei et al., 2006a; Thompson et al., 2008; Iglesias et al., 2009). The opening of Panx1 channels has been induced by various experimental conditions, including activation of purinergic receptors, stretch, high intracellular calcium, and membrane depolarization (Bao et al., 2004; Locovei et al., 2006b; Pelegrin and Surprenant, 2006). Panx1 is insensitive to variations in extracellular calcium concentrations over a wide range (Bruzzone et al., 2005) and opens at negative resting potential in response to mechanical stress and at micromolar increase of intracellular calcium (Bao et al., 2004; Locovei et al., 2006a). In conjunction with the sensitivity of Panx1 to extracellular ATP through purinergic receptors of the P2Y and P2X type (Locovei et al., 2007), it has been postulated that a prime function of Panx1 channels are as ATP release conduits mediating extracellular propagation of Ca^2+^ waves (Locovei et al., 2006a,b). Astrocytes propagate Ca^2+^ waves (Cornell-Bell et al., 1990; Arcuino et al., 2002) and have been suggested to be an important mechanism in neuronal – glial signal transmission (Nedergaard et al., 2003; Newman, 2003; Volterra and Steinhauser, 2004).

It has been proposed that Panx1 channels are involved in pathways activated upon a strong stimulus, such as inflammation (Kanneganti et al., 2007; Silverman et al., 2009), ischemia (Thompson et al., 2006), or myocardial infarction (Dolmatova et al., 2012). In keeping with the hypothesis that Panx1 channels open only upon a stimulus, Panx1 channels are constitutively closed (Bao et al., 2004; Bruzzone et al., 2005). Panx1 channels (pannexons) lack sensitivity to extracellular Ca^2+^, an important property, as Panx1 channels will not be open under physiological ionic conditions. In neurons and possibly astrocytes, Panx1 may contribute to novel forms of synaptic and non-synaptic communication and Ca^2+^ wave propagation (MacVicar and Thompson, 2010). In mammalian skin, Panx1 plays a key role in keratinocyte differentiation. Furthermore, in the cardiac, nervous, and immune systems, Panx1 activation is implicated in ischemic, excitotoxic, and ATP-dependent cell death such that Panx1 coupling with purinergic receptors triggers an inflammasome mediated response (Kanneganti et al., 2007; Pelegrin et al., 2008; Silverman et al., 2009). Reactive astrocytes immediately bordering an abscess exhibited open Panx1 channels during the acute inflammatory period, which dissipated as the infection evolved (Karpuk et al., 2011). Additionally, P_2_X_7_ receptor activation induces Panx1 channel opening in astrocytes (Scemes et al., 2007; Iglesias et al., 2009; Suadicani et al., 2012). Panx1 channel activity has been implicated recently in inflammasome formation in cultured neurons and astrocytes (Silverman et al., 2009). The latter data suggest a crucial role for Panx1 in several cell death pathways in the nervous system (Bargiotas et al., 2009; MacVicar and Thompson, 2010), although a recent Panx1 KO mouse showed no morphological changes in brain slices by histological analysis or changes in IL-1β from macrophages (Bargiotas et al., 2011). The authors stated in the on-line supporting information that five out of six commonly used anti-Panx1 antibodies tested on KO mouse tissue in Western blots were “non-specific,” however no original data detailing protein specificity from these Western blots or immunofluorescence imaging was provided. It is possible that in actuality some tissues of that particular KO mouse still contain Panx1 protein or again, Western blots are not the only diagnostic to be trusted for Panxs.

### Generating a Panx1 antibody tool-kit

The four antibodies used in this study performed well in cell culture by labeling exogenous protein with an independent tag and showed a consistent and similar banding pattern on Western blots. In generating or testing antibodies against anti-Panx1 peptides, we used several standard criteria for a “good” antibody. One quality for a good Panx1 antibody generated was the ability to recognize recombinant protein containing an appended independent tag when expressed in tissue culture cells such that an antibody against the tag overlapped with the Panx1 staining. As important, was that the Panx1 labeling patterns in exogenously expressing cultured cells would be similar to those found in endogenously expressing Panx1 cells. In our case, endogenously expressing Panx1 cell staining was very similar to transfected cells (Boassa et al., 2007). Another criterion was that in model systems, antibodies would recognize recombinant protein and endogenous protein on Western blots in a manner consistent with their expected molecular size and/or post-translational modifications. As a negative control, elimination of the primary antibody would abolish signal on Western blots of tissue culture cells and/or on tissue. For recombinant Panx1 in cells, Western blots of parental cell lines provide a critical negative control. We showed using Panx1 KO mice as a negative control (Figures 3B,C) that these four antibodies recognize Panx1 at its expected sizes as well as some sizes that are not yet explainable. There are some non-specific bands on some of the Western blots that do not disappear in the knock out tissue lysates consistently, as they are only present in some tissue samples. It is worth noting that Qiu et al. (2011) and Santiago et al. (2011) used the Ck4515 antibody to validate lack of expression in a Panx1 KO. Notably, two recent publications (Burns et al., 2012; Lohman et al., 2012) used immunoblotting, immunohistochemistry, and RT-PCR methods to cross-validate expression patterns in cerebral vasculature. However, it is important to remember that RT-PCR methods assay for mRNA expression, which can be different from protein expression. For example, Lohman et al. (2012) demonstrated that HEK293 cells, a cell line that has been documented in several studies as being negative for Panx1 expression, has residual Panx1 mRNA but no protein expression as assayed by Western blots and immunohistochemical imaging.

This work focused on imaging Panx1 expression in select areas of the rat brain and compared their cellular localizations. Although Panx1 antibodies have been criticized for lack of specificity, this comparative labeling study takes into account the differences between antibody epitopes and labeling requirements and utilizes their unique qualities. Uncertainty in the antibodies currently available (both privately and commercially) is due to various reasons. It is hard to find a single antibody that can perform well by recognizing the Panx1 protein under all Western blot and immuno-microscopy conditions, which has been shown for other protein targets as well especially in brain tissue (Herkenham et al., 2011). Another reason for uncertainty about Panx1 antibodies has been the failure to fully characterize, at the protein level, the original report of a Panx1 KO mouse that was generated several years ago (Anselmi et al., 2008). Now additional Panx1 KO mouse lines have been generated by various techniques and so far they all perform differently under these rigorous tests by Western blot. Because of this discrepancy and the difficulty in brain tissue preservation, there are few published cellular level images of Panx1 protein expression in brain and all have limited fields of view (Ray et al., 2006; Zappala et al., 2006; Zoidl et al., 2007; Karpuk et al., 2011). We performed these experiments in rat brain because no non-specific cross-reaction would occur due to labeling the same species that the antibodies were generated against (a mouse against mouse reaction).

### Comparison of protein labeling to *In situ* hybridization studies

The ability to combine gross structural imaging with high-resolution microscopy on a large scale significantly advances our ability to detect and analyze multiple brain regions. The trade-off between resolution and field of view leads to targeted investigations limited to regions of the brain that are determined to be heavily labeled and well studied while ignoring areas that may be smaller or more subtly labeled. *In situ* hybridization provided a first estimate of where pannexins are located (Baranova et al., 2004; Vogt et al., 2005; Weickert et al., 2005). *In situ* hybridization provides a first approach to assembling a protein expression map in tissues because it is easier and faster to generate riboprobes than antibodies. However, proteins in the nervous system can be very far removed from where the genes are expressed; thus, *in situ* hybridization investigations give only a limited view of expression patterns. Correlation between the two types of staining can be difficult, because antibodies do not always label the protein in the cell body, leading to difficulties in ascertaining the identity of labeled axons and dendrites.

For the most part, our antibody labeling across brain domains are similar to those obtained with *in situ* hybridization. Strong staining has been observed in the cerebellum, hippocampus, olfactory bulb, and thalamus (Ray et al., 2005; Vogt et al., 2005). In the cerebellum (Vogt et al., 2005), found that all layers showed signal but it was highest in Purkinje cells, Golgi cells, and deep cerebellar nuclear cells. In the olfactory bulb, we found that the strongest signal was in the mitral cell layer and AOB. Vogt et al. (2005) showed that some labeling occurred in the granule cell layer of the olfactory bulb. The hippocampus contained high Panx1 expression in the dentate gyrus and all CA regions, pyramidal cells, stratum oriens, radiatum, and lacunosum-moleculare. (Vogt et al., 2005) found that co-labeling with GFAP and NeuN antibodies with riboprobes highlighted that neurons were labeled but astrocytes were not. In particular, Vogt et al. (2005) found that GABAergic interneurons and pyramidal neurons showed significant Panx1 staining. In our study, we found that, while there were consistencies between the four antibody labels, the subcellular distribution could vary as shown in the pyramidal neurons of the hippocampus. We observed Panx1 staining in astrocytes using all of the polyclonal antibodies including CkDia, although initial characterization of this antibody by Zappala et al. (2006) did not include descriptions of astrocytic staining. It should be noted that Panx1 expression in astrocytes was recently demonstrated *in vivo* both functionally and by immunohistochemistry (Karpuk et al., 2011; Santiago et al., 2011) and in Western blot analysis of cultured astrocytes (Huang et al., 2007; Iglesias et al., 2009; Silverman et al., 2009; Suadicani et al., 2012). High Panx1 RNA expression was documented from staining of the thalamic reticular nucleus (Ray et al., 2005), while we find protein labeling of sporadic cells in this region with each antibody that was used for staining.

### Consistent and variable features between the four antibody labeling patterns

A major goal of this study was to determine subcellular patterns of expression across the different brain regions and search for consistent patterns between the four antibodies. Each of these antibodies has a unique epitope with only two (CkDia and Rb57) of them overlapping. As such, these two antibodies had similar domain and subcellular labeling patterns, in that they tended to highlight the neuronal cell bodies as well as astrocytes, and endothelial cells in most of the brain regions we imaged. Some images are suggestive of plasma membrane staining, particularly with the Mo503 antibody. However, electron microscopy of these slices will be necessary to unequivocally determine this, since it is sometimes difficult to determine proteins or protein complexes that lie just underneath or within the plasma membrane. The Ck4515 antibody, a very commonly used antibody within the pannexin field, has an expression pattern that is closer to the CkDia and Rb57 labeling patterns than the Mo503 labeling. Our results in hippocampus with the Ck4515 antibody were similar to those of Santiago et al. (2011) who used an antibody generated against the same peptide as Ck4515 and showed labeling in neurons, astrocytes, and perivascular astrocytic endfeet in hippocampal area CA1. That study also showed no non-specific labeling in KO mouse tissue.

### Modifications to Panx1 gene expression that could impact expression patterns

Other considerations in understanding, Panx1 labeling include post-translational modifications or differentially translated Panx1 that could complicate various banding patterns that were unanticipated from tissue culture control experiments. Using the same tissue lysate, the Western blot protein patterns varied depending on the antibody and presented a more complex situation than tissue culture cells. Our Western blots of the same rat and mouse brain lysates varied from two to eight major bands depending on the probing antibody. In other published studies, bands were shown at ∼43, ∼52, and ∼96 kDa in Western blots of mouse and rat brain lysates (Wang et al., 2009) while five bands corresponding in approximate size to exogenously expressed Panx1 protein were recently shown in rat brain lysate by Western blot (Kienitz et al., 2011).

Several factors may influence the number of bands seen on Western blots. Glycosylation results in having multiple bands although the number can range depending on the tissue and cell type (Boassa et al., 2007; Penuela et al., 2007). Furthermore, these glycosylated species were shown to give rise to spatial separation of the fully glycosylated GLY2 in the plasma membrane and the GLY0 and GLY1 isotypes in intracellular compartments such as the ER. Recently, Panx1 has been shown to have two potential caspase cleavage sites of which one site was cleaved by caspases 3 and 7 (Chekeni et al., 2010). Either of these two sites, cleaved separately or in combination in brain tissue, would cause differential recognition of the resulting protein by these four antibodies (Table 3, see sites in Figure 1). Panx1 has also recently been shown to be expressed as three additional splice variants in the pituitary gland (Li et al., 2011) that would also give rise to differential recognition of Panx1 species of 48 kDa by all antibodies for the full-length (“Panx1a” splice variant), 40 kDa for Ck4515, and Mo503 antibodies for the “Panx1c” splice variant or 35 kDa recognized by CkDia, Rb57, and Mo503 for the “Panx1d” splice variant. In addition, it is important to note that glycosylation of several of these Panx1 species will affect the experimentally measured monomer size if the Panx1 species retains the N254 glycosylation site. Lastly, while inhibitors of proteolytic enzymes are typically included in lysate protocols and prevent gross damage, proteolytic cleavage may still occur during the dissection and homogenization process.

**Table 3:** **Differential recognition of predicted fragments resulting from caspase cleavage of Panx1**.

	Ck4515	CkDia	Rb57	Mo503
Full-length Panx1	48*	48*	48*	48*
Caspase site A cleavage	30*	18	18	18
Caspase site B cleavage	6	42*	42*	42*
Double caspase cleavage at both sites	6	18	18	18

These antibodies are predicted to recognize differently sized fragments (expressed in kDa) of the Panx1 protein after caspase cleavage (see Figure 1 for schematic). * = fragment containing the *N*-glycosylation site.

The complex labeling patterns we see may be a reflection of different cellular and subcellular localizations, where each antibody is highlighting unique subpopulations of accessible Panx1 epitopes of this dynamically expressed and processed protein. For example, in many cell types the Mo503 antibody preferentially labels dendrites and not cell bodies, while the other antibodies often highlight the cell bodies. The mouse antibody is unique in that it was generated against part of the N-terminus. Our tissue labeling results are consistent with tissue culture systems where Panx1 is localized to both the plasma membrane and intracellular membrane compartments. It is important to note that some tissue culture cells such as bEnd3 vascular endothelial cells show only intracellular immunofluorescence that correlated with the appearance of only the GLY0 band on Western blots (Boassa et al., 2007). At the present time, we believe a more in-depth analysis of Panx1 gene and protein modifications will be necessary to fully interpret the bands we see on our Western blots.

### Neuroinformatics resources and tools for deciphering Panx1 protein expression

Although we cannot conclusively establish localization of pannexin protein because of the variability between antibodies, tissues, and assay systems, we believe that this paper illustrates several best practices that should be employed in all immunolabeling studies to make it easier to compare findings and to ensure that problematic information is not suppressed during the publication process, but rather exposed so that the community has accurate information (MacArthur, 2012). As a first step, we provided all identifying information for each antibody (vendor, catalog#, clone ID, and Antibody Registry URI). The Antibody Registry is a web resource that issues a stable, traceable, permanent identifier for an antibody created by commercial vendors or individual labs so that a researcher can easily find the antibody used in a scientific paper and trace that antibody back to the creator. Often, manufacturers have more than one antibody to the same protein, and it is important that the catalog number be included for all reagents. However, commercial vendor IDs may not be stable over time, as vendors go out of business or sell their product lines. For this reason, the Neuroscience Information Framework (NIF^4^), a project designed to make it easier to identify resources of relevance to neuroscience (Gardner et al., 2008), created the Antibody Registry^5^. The Antibody Registry currently contains >900,000 antibodies, largely from commercial sources. Each is given its own unique accession number, much like a gene sequence in GenBank that can be used to uniquely identify an antibody. Currently, there are 16 commercially available Panx1 antibodies listed on the Antibody Registry. There are many more that were privately generated and used in publications such Bargiotas et al. (2011); Huang et al. (2007). Again, we include the identifiers for our four antibodies in the materials and methods of the paper, so that text mining algorithms and authors can identify the usage of these antibodies without ambiguity. If such identifiers were routinely used, researchers could pull out all papers that use a particular reagent. More importantly, if problems like specificity come to light, notifications can be placed on those papers whose results may need to be re-analyzed or re-interpreted.

A second best practice is to publish all the data from a paper rather than selecting only the best exemplars. In our case, we show all Western blots for all antibodies and provide access to the full resolution brain maps via the CCDB. We believe that for novel proteins or those for which it is difficult to develop good probes, the type of survey study we publish here provides a public platform and forum, for evaluating the specificity of the reagents and ensuring that problems with these reagents are widely disseminated rather than suppressed. Often times, such knowledge is known by experts in the field, but it may take years for this knowledge to percolate to the wider community (MacArthur, 2012), leading to continued use of faulty reagents during this time.

Finally, ensuring that papers are published in open access journals or deposited within Pub Med Central or made available through a pre-print service is critical, to ensure that search engines can gain access to the Materials and Methods section of the paper.

Thus, with this study we attempt to promote explorations that move beyond the question of why peptide specific antibodies do and do not label in brain tissue and instead examine how they work differently, the complexity of the biological system and how to interpret further imaging experiments. Our imaging strategy provides a novel approach such that we release the datasets (montages, associated metadata, and “raw” images) in the CCDB in their full size, resolution and complexity rather than presenting only down-sampled and/or redacted images that one typically finds in publications. By doing so, this unrestricted available data rich resource promotes further data mining and explorations by the public. Other resources like the Allen Brain Atlas and GENSAT may be useful for interpreting our datasets. The Allen Brain Atlas, a publicly available image database for *in situ* hybridization images of mouse brain and associated tools for information extraction and visualization (Lein et al., 2007), is very useful as a first step for correlating with gene expression hotspots. The Gene Expression Nervous System Atlas (GENSAT) project uses bacterial artificial chromosome (BAC) vectors in combination with transgenic mouse to EGFP tag proteins in the CNS for light microscopy (Gong et al., 2003). While, transgenic Panx1-EGFP is not yet available in this on-line digital atlas and there is the risk that recombinant Panx1-EGFP may not traffic the same as wild type protein, the GENSAT database can be used to correlate with other proteins in the database for overlap in expression patterns. The 18 montages from this study significantly increase the number and expanse of published images of Panx1 labeling in brain. However, no comprehensive protein expression atlas yet exists for the rodent brain, equivalent in breadth to the Allen Brain Atlas. As we see here, antibody labeling is highly variable and dependent on specimen preparation protocols, even with antibodies to the same protein. Our study points out the importance of thorough characterization and unambiguous identification of the antibody reagents used to report findings. Currently, identification of antibody reagents from published studies is very difficult, as most authors do not provide enough detailed information (e.g., catalog number) to identify the exact antibody used (Bandrowski et al., submitted). The Neurosciences Informatics Framework (NIF^6^) has created a large antibody registry where antibodies receive a uniform resource identifier (URI) that can be used to reference these reagents in papers (see Table 1,^7^). We encourage the use of these numbers in their papers.

In summary, because this situation occurs more frequently than is documented, we recommend the following protocol: (1) Entire data sets should be made available on-line, (2) Reagents need to be specified, (3) In order to clarify who else uses these reagents, each antibody should have a unique URI, like those utilized by NIF. Reproducibility of reagents and protocols are critical. A recent editorial in Nature highlighted significant reproducibility issues in clinical drug trials because of insufficient or selective reporting (Begley and Ellis, 2012). Here we embrace the difficulties by publishing in a form that is fully vetted, showing all the data. By being able to manipulate the levels via the WIB, researchers can form their own opinion of the data. We encourage readers to leave comments via the Frontiers website.

## Conflict of Interest Statement

The authors declare that the research was conducted in the absence of any commercial or financial relationships that could be construed as a potential conflict of interest.

## Footnotes

^1^https://confluence.crbs.ucsd.edu/display/ncmir/Mosaic+Plugins+for+ImageJ

^2^http://openccdb.org/software/index.shtm

^3^http://ccdb.ucsd.edu

^4^http://neuinfo.org

^5^http://antibodyregistry.org

^6^http://neuroinfo.org

^7^http://antibodyregistry.org/antibody17/antibodyform.html?gui_type = advanced

## Abbreviations

CNS, central nervous system; Panx1, pannexin1.
